# Clinical Feasibility and Mechanical Reliability of a Modified Functional Articulating Hip Spacer Incorporating a Cemented Dual Mobility Bearing Metal Liner

**DOI:** 10.3390/jcm15062309

**Published:** 2026-03-18

**Authors:** Sun-hyung Lee, Soong Joon Lee

**Affiliations:** 1 Department of Orthopaedic Surgery, Seoul Metropolitan Government-Seoul National University Boramae Medical Center, Seoul 07061, Republic of Korea; dinggu32@snu.ac.kr; 2 Department of Orthopaedic Surgery, Seoul National University College of Medicine, Seoul 03080, Republic of Korea

**Keywords:** periprosthetic joint infection, native hip joint infection, PROSTALAC, dual-mobility bearing

## Abstract

**Background**: Periprosthetic joint infection and native hip infections often require staged surgical intervention due to extensive bone and soft tissue destruction. This study evaluates the clinical feasibility and mechanical reliability of a modified functional articulating hip spacer (FAHS) incorporating a cemented dual-mobility-bearing (DMB) metal liner. **Methods**: We retrospectively reviewed the cases of 20 patients who underwent a DMB-incorporated FAHS between March 2018 and December 2019. The technique involved cementing a DMB metal liner directly into the prepared acetabulum without a standard outer shell. Successful clinical outcome was defined as either transition to second-stage total hip arthroplasty (THA) or stable spacer retention, the latter including cases with definitive eradication or symptom-controlled chronic suppression therapy. Infection eradication required the clinical absence of infection for at least twelve months following the cessation of antimicrobial therapy. Construct-related mechanical complications and radiographic parameters were also analyzed. **Results**: The mean follow-up was 23.5 months, ranging from 6.0 to 62.6 months. Successful clinical outcome was achieved in 17 patients (85%), with seven (35%) transitioning to second-stage THA and ten (50%) opting for spacer retention. Within the retention group, seven achieved definitive eradication while three were maintained under chronic suppression therapy. Construct integrity was maintained in 80% of the cohort. Mechanical complications included two dislocations (10%) and two implant failures (10%). Radiographic analysis showed higher inclination and anteversion angles of the metal liner in the dislocation cases. **Conclusions**: The off-label use of DMB-incorporated FAHS represents a feasible option with acceptable mechanical performance in selected cases of PJI and native hip joint infection. However, as mechanical complications cannot be fully prevented, meticulous surgical techniques and careful patient selection remain essential.

## 1. Introduction

Periprosthetic joint infection (PJI) and native hip joint infections are among the most challenging conditions encountered in orthopedic surgery. Both can result in extensive bone and soft tissue destruction, necessitating staged surgical intervention. Historically, these complex infections have been managed using a “one-size-fits-all” approach, with two-stage exchange arthroplasty [1,2]. The two-stage revision protocol remains the gold standard for managing these infections, combining aggressive debridement with delayed reimplantation after infection eradication [3,4,5]. During the interim period, a PROSTALAC (prosthesis with antibiotic-loaded acrylic cement) construct is often used to maintain joint space, allow limited mobility, and deliver local antibiotics [6,7]. However, recent clinical evidence suggests that this conventional paradigm is no longer universally applicable, as it imposes a substantial physiological and economic burden on patients, particularly the elderly or those with significant comorbidities [1,2]. Consequently, the paradigm is shifting toward more individualized treatment strategies, leading to the emergence of the 1.5-stage exchange as a viable alternative. This strategy utilizes a functional articulating hip spacer (FAHS) as either a reliable bridge to staged revision or a definitive destination spacer for patients who are poor candidates for further major surgical procedures [8,9,10].

The primary clinical advantage of an FAHS lies in its ability to preserve joint mobility and facilitate early mobilization, which is essential for preventing soft tissue contracture and supporting patient recovery during the interim period [11]. A well-recognized commercial option for this purpose is the PROSTALAC^®^ Hip System (DePuy-Synthes, Warsaw, IN, USA). While this system has demonstrated success in clinical practice, its clinical application is often limited by high procedural costs and restricted regional availability, especially in healthcare settings that lack dedicated pre-made articulating spacer kits [12]. These constraints necessitate the development of practical and mechanically robust alternatives that utilize standard orthopedic implants to maintain stable articulation and enable ambulation and transfers during the interim period.

In healthcare environments where standardized articulating systems like the PROSTALAC^®^ are not readily accessible, an innovative approach is required to manage hip joint infections with severe joint destruction. To address this clinical gap, we developed a modified FAHS construct by incorporating a standard dual mobility bearing (DMB) metal liner cemented directly into the acetabulum. This modified construct leverages the high jump distance and inherent mechanical stability of the DMB system [13,14,15,16] and was designed to provide stable articulation while mitigating dislocation risk and maintaining construct integrity in complex infection scenarios with significant acetabular bone loss. Importantly, the present investigation primarily focuses on the feasibility and mechanical behavior of this off-label construct rather than on comparative effectiveness against established spacer systems.

We hypothesized that cementing a DMB metal liner directly into the acetabulum could provide acceptable joint stability and function as part of a modified FAHS construct in selected complex infection cases. Therefore, the purpose of this study was to evaluate the clinical feasibility and mechanical reliability of this DMB-incorporated FAHS, evaluating its performance both as a bridge to staged revision and as a destination spacer for long-term retention in PJI and native hip infections.

## 2. Materials and Methods

This retrospective study was approved by the Institutional Review Board of our institution (IRB No. 10-2025-27), with a waiver for informed consent. From March 2018 to December 2019, 148 patients underwent surgical treatment for either PJI or native hip joint infection, defined by MSIS criteria [17] at our institution. Among them, 22 patients received a DMB-incorporated FAHS. After excluding two patients lost to follow-up for less than 6 months, 20 patients were included in the final analysis.

The DMB-incorporated FAHS was selectively applied in patients who demonstrated substantial acetabular cartilage loss, significant acetabular bony defects, or an anticipated need for prolonged spacer retention due to medical frailty or complex infection conditions. These indications were determined intraoperatively based on the extent of bone and cartilage destruction and the likelihood of expected extended interim spacer retention. All procedures were undertaken with the intent of a staged approach in which the DMB-incorporated FAHS served as an interim spacer before second-stage total hip arthroplasty.

All surgeries were performed with the patient in the lateral decubitus position using a direct lateral approach. In cases of PJI, all previous implants were removed, with or without an extended trochanteric osteotomy, depending on the presence of stem loosening. In native hip infection cases, the femoral head was osteotomized and removed. Thorough debridement of infected or necrotic tissue was performed in all cases. The femoral canal was prepared using flexible reamers, and the acetabulum was sequentially reamed to expose healthy, bleeding cancellous bone. The femoral component was prepared intraoperatively using antibiotic-loaded cement molded around a previously sterilized, size-matched core stem retrieved from prior revision cases. For the acetabular component, a metal liner from a dual mobility system (Zimmer-Biomet, Warsaw, IN, USA or Stryker, Mahwah, NJ, USA) was cemented directly into the prepared acetabulum using the same antibiotic-loaded cement. No surface modification such as roughening or drilling was performed to avoid compromising the structural integrity of the liner, given the off-label nature of its use. The standard outer metal shell of the DMB was not used. After the cement cured, a polyethylene insert was coupled to the metal liner, and reduction was performed to complete the construct.

Postoperatively, intravenous antibiotics were administered for 6 to 8 weeks, followed by oral antibiotics, as determined in consultation with infectious disease specialists. Second-stage total hip arthroplasty (THA) was considered in patients who achieved infection eradication and expressed a desire for definitive reimplantation, typically after a minimum of 3 months.

Patients were followed clinically and radiographically. Clinical records were reviewed to evaluate construct-related mechanical complications (including dislocation, cement failure, and liner dissociation), the need for reoperation, and whether the DMB-incorporated FAHS was retained or revised. Radiographs were assessed for implant loosening, component position, and mechanical integrity. Inclination and anteversion angles of the cemented metal liner were measured using standard anteroposterior and cross-table lateral views.

The successful clinical outcome was evaluated based on a multidimensional framework adapted from the Delphi consensus of Diaz-Ledezma et al. [18]. While the original consensus prioritizes complete infection eradication, we defined a successful clinical outcome more broadly to reflect the therapeutic goals of functional joint preservation and the avoidance of additional surgery. Specifically, a successful clinical outcome was categorized into two groups: the successful transition to second-stage THA and the stable retention of the DMB-incorporated FAHS. For patients in the staged revision group, success was defined by the eradication of infection followed by a successful reimplantation surgery without subsequent recurrence. For patients in the spacer retention group, success was defined as the maintenance of a functional joint without the need for additional debridement or resection. Within this retention group, we further distinguished between cases with definitive infection eradication and cases under chronic suppression therapy. Definitive eradication was strictly defined as the absence of clinical signs such as persistent pain or a sinus tract for at least twelve months after the cessation of all antimicrobial therapy. Chronic suppression therapy was defined as the absence of active infection symptoms while continuing long-term oral antibiotic therapy.

Given the small sample size and low event frequency, all statistical analyses were exploratory, and any between-group comparisons should be interpreted descriptively.

For the visualization of individual clinical courses and the construction of the swimmer plot presented in Figure 3, Gemini 3.0 (Google, Mountain View, CA, USA) was utilized. The authors manually verified the generated output against the original clinical database to ensure accuracy and remain fully responsible for the final visual representation.

## 3. Results

A total of 20 patients were included in the final analysis. The mean follow-up duration was 23.5 months (range, 6.0–62.6 months). The demographic and clinical characteristics of the patients are summarized in Table 1.

### 3.1. Clinical Outcomes

**Figure 1:**
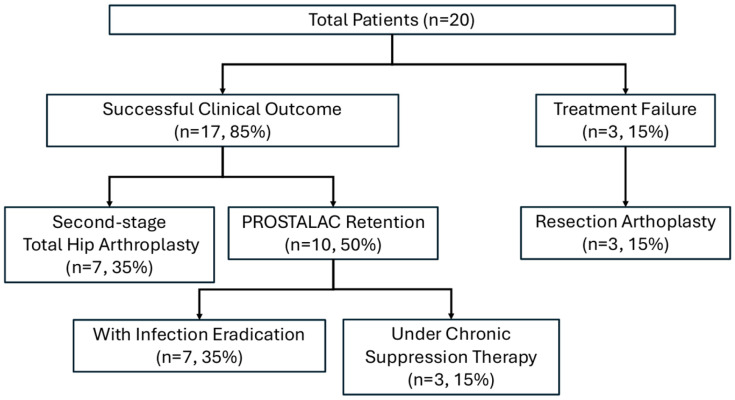
Flowchart illustrating the clinical outcomes. Successful clinical outcomes were achieved in seventeen patients (85%), including seven (35%) who transitioned to staged revision and ten (50%) who retained the spacer. Within the retention group, seven patients (35%) achieved definitive eradication while three (15%) were maintained under chronic suppression. Three treatment failures (15%) necessitated resection arthroplasty due to persistent infection.

**Figure 2:**
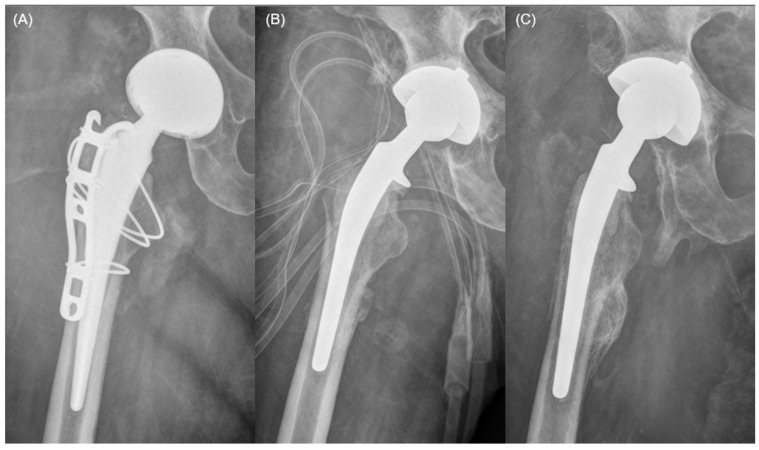
Serial radiographs of a patient who retained the dual-mobility bearing (DMB)-incorporated functional articulating hip spacer (FAHS) over long-term follow-up. (**A**) A 60-year-old woman developed PJI following primary total hip arthroplasty (THA) performed at an outside hospital. (**B**) After removal of all implants, a DMB-incorporated FAHS was implanted. (**C**) At 39.2 months postoperatively, the patient remained infection-free and continued to retain the DMB-incorporated FAHS without undergoing second-stage THA, per patient preference.

**Figure 3:**
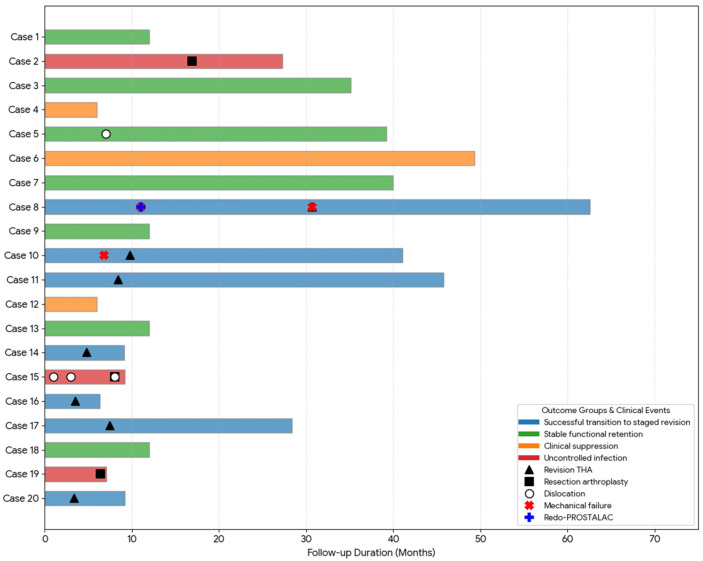
Swimmer plot of individual clinical courses and key events. Each horizontal bar represents the follow-up duration, with color indicating the final clinical outcome groups. Symbols identify key surgical and mechanical events, including staged revision, resection arthroplasty, and various mechanical complications as specified in the figure key.

At the final follow-up, 17 patients (85%) achieved a successful clinical outcome, categorized into three distinct clinical pathways (Figure 1). Infection status was reported separately as definitive eradication or clinical control under chronic suppression therapy. The first pathway involved successful bridging to a second-stage THA, achieved by seven patients (35%) who underwent definitive revision surgery after confirmed infection eradication. Notably, two patients in this group reached the goal despite requiring intermediate interventions for mechanical issues. The second pathway represented stable retention with infection eradication, where seven patients (35%) opted to retain the DMB-incorporated FAHS as a long-term solution. These patients reported satisfactory joint function and preferred to avoid further major surgical procedures (Figure 2). The third pathway consisted of three patients (15%) maintained under clinical control via chronic suppression therapy with oral antibiotics. Within this group, two patients remained clinically infection-free after discontinuing antibiotics but were conservatively classified as receiving suppression therapy because their follow-up duration after antibiotic cessation had not yet reached the predefined 12-month threshold required for the formal definition of eradication. The remaining three patients (15%) were considered treatment failures and required resection arthroplasty due to persistent and uncontrolled infection. The pathogens identified in the three failure cases were Candida albicans (*n* = 2) and MRSA (*n* = 1). The individual clinical course and the specific timeline of transitions between different treatment pathways for all 20 patients are visually summarized in the swimmer plot (Figure 3). Outcomes were additionally summarized by diagnosis (PJI vs. native hip infection) in a descriptive manner (Table 2), without formal between-group comparisons.

### 3.2. Mechanical Reliability and Construct-Related Complications

Sixteen patients (80%) maintained construct integrity without any implant-related complications throughout the interim or retention period. Mechanical complications were observed in four patients (20%) and were categorized as dislocation and implant failures.

Dislocation occurred in two patients (10%). One patient (Case 5) experienced a single dislocation event that was successfully managed with closed reduction, subsequently allowing for long-term retention of the DMB-incorporated FAHS (Figure 3). In contrast, another patient (Case 15) who suffered from uncontrolled infection experienced recurrent dislocations, eventually necessitating resection arthroplasty (Figure 3).

**Figure 4:**
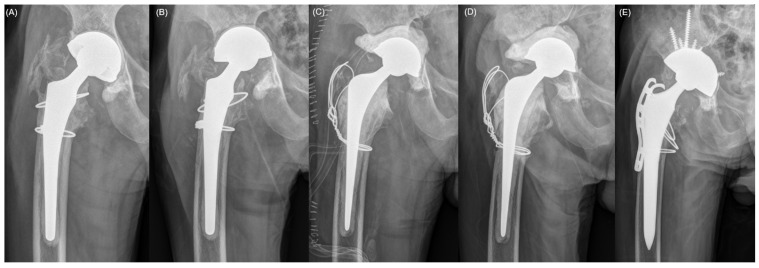
Recurrent cement mantle failure and subsequent revision in a 78-year-old male. (**A**) Immediate postoperative radiograph following the initial dual-mobility bearing (DMB)-incorporated functional articulating hip spacer (FAHS) implantation for periprosthetic joint infection. (**B**) Radiograph demonstrating the first mechanical failure characterized by medial displacement of the cement mantle into the acetabular defect. (**C**) Radiograph after a redo-FAHS procedure performed to restore joint continuity. (**D**) Radiograph showing a second cement mantle failure after the redo surgery. (**E**) Final radiograph following definitive revision total hip arthroplasty after infection eradication was achieved.

Implant failure occurred in two patients (10%). One patient (Case 8) experienced cement failure and underwent a redo-FAHS procedure; however, another cement failure after infection eradication ultimately led to a second-stage THA (Figure 3). Another patient (Case 10) experienced dissociation between the cement mantle and the metal liner, which was managed by retaining the DMB-incorporated FAHS until infection eradication was achieved, followed by a successful second-stage revision (Figure 3 and Figure 4). Patients (Case 6) who retained the DMB-incorporated FAHS for an extended duration demonstrated stable mechanical performance without evidence of spontaneous loosening or hardware-related adverse reactions for up to 49.3 months (Figure 3).

### 3.3. Radiologic Assessment

Radiographic analysis indicated that 45% (9/20) of the components were positioned within the Lewinnek safe zone [19]. For the entire cohort, the mean inclination angle was 40.0° ± 10.2° and the mean anteversion angle was 19.6° ± 11.2°. The two dislocation cases showed higher inclination angles than the non-dislocation cases (49.1° ± 4.5° vs. 38.9° ± 10.2°), and anteversion angles were also modestly higher (22.3° ± 0.1° vs. 19.3° ± 11.8°). Because dislocation events were infrequent, subgroup comparisons should be regarded as exploratory.

## 4. Discussion

This study evaluated the clinical feasibility and mechanical reliability of the DMB-incorporated FAHS. Our findings demonstrate that this DMB-incorporated FAHS construct achieved an 85% successful clinical outcome rate, effectively serving as either a stable bridge to staged revision or a destination spacer for long-term retention, with infection endpoints reported separately as definitive eradication or clinical control under chronic suppression therapy. The potential advantage of this approach lies in its ability to provide improved mechanical stability and preserve joint continuity, particularly in complex cases of PJI and native hip joint infections with significant acetabular destruction.

The conceptual shift from a “one-size-fits-all” two-stage exchange toward a more individualized 1.5-stage strategy is a key theme in contemporary infection management. As highlighted by Hannon et al. [2], the traditional gold standard of staged revision can impose prohibitive physiological and economic burdens on elderly or medically frail patients. In our cohort, 50% of the patients who achieved definite eradication of infection opted for the long-term retention of the DMB-incorporated FAHS rather than proceeding to a second-stage THA. This high retention rate supports the potential role of the DMB-incorporated FAHS as a “destination spacer,” providing sufficient joint function and stability to satisfy patients’ daily living requirements while avoiding the morbidity associated with further major surgery.

The mechanical rationale for incorporating a DMB metal liner instead of a traditional cemented polyethylene liner or a unipolar cement head centers on the significantly increased jump distance and inherent stability of the dual mobility system [13,14,15,16]. Traditional cement-on-bone articulations are often plagued by pain, pelvic bone wear, and superior migration, especially in the presence of pre-existing acetabular defects [20,21,22]. By creating a formal mechanical articulation with a DMB liner, we intended to mitigate these issues. A noteworthy observation from our study was the relatively high retention rate of the DMB-incorporated FAHS after infection eradication was achieved. Specifically, half of the patients with eradication of infection opted to retain the spacer rather than proceed to second-stage total hip arthroplasty. Patients maintained stable implant fixation without mechanical complications, even up to 49.3 months of follow-up. The average follow-up period for patients who retained the DMB-incorporated FAHS was 20.0 months (range, 6.0–49.3 months). Although this study did not evaluate pain or functional outcomes due to its retrospective nature, this finding suggests acceptable interim function and clinical tolerability of the DMB articulation.

Despite the theoretical stability advantages of dual mobility, dislocation remained possible in this cohort. Our observed dislocation rate (10%) aligns with previously reported dislocation rates of conventional PROSTALAC spacers (10% to 20%) [20,23,24,25,26]. While the exact cause of dislocation is likely multifactorial, the cases with dislocation had greater inclination and anteversion angles of the metal liner compared to cases without dislocation. These results, although exploratory, underscore that proper positioning of the liner remains critical, even with DMB systems. However, intraoperatively, bony landmarks are often obscured due to infection-related bone loss and soft tissue debridement, and anatomical orientation is difficult to assess during cementation. In this study, only 9 of 20 metal liners (45%) were within the Lewinnek safe zone [19]. This reflects the technical demands of the procedure and highlights the importance of meticulous positioning. Therefore, even with dual-mobility articulation, careful liner positioning remains essential, and mechanical complications can still occur.

The primary mechanical concerns with this off-label approach involved cement and liner fixation durability. Implant failure was observed in two patients (10%). These failures comprised a total of three distinct mechanical events. Specifically, one patient experienced two separate instances of cement mantle failure, including a recurrence that occurred following a redo-FAHS procedure. The other patient suffered from a single event characterized by dissociation between the metal liner and the cement mantle. Both cement failures occurred medially, with the cement mantle displaced into cavitary acetabular defects (Figure 4). This was likely due to insufficient bony containment and the inability to provide adequate counterforce against compressive loading during weight-bearing. Extensive medial bone loss was observed intraoperatively and confirmed on postoperative radiographs, suggesting that large uncontained defects in the medial wall may cause predisposition to cement failure. These implant failures represent clinically meaningful events and underscore the necessity of a thorough assessment of acetabular bone stock before adopting this technique. These findings indicate that this technique may not be suitable in the presence of substantial medial acetabular defects without additional structural support. Accordingly, given the apparent vulnerability in uncontained medial defects, careful intraoperative assessment of medial wall integrity and overall acetabular containment should guide case selection.

Although cement is not an adhesive in the strict sense, the construct is primarily exposed to compressive loading during weight-bearing, which may favor interface stability.

In typical daily activities, tensile or extraction forces that might dislodge the cemented liner are rare. Instead, the prosthesis primarily experiences compressive loading during weight-bearing, which is more favorable for maintaining the mechanical stability of the cemented interface. Furthermore, these patients usually have a limited range of motion and reduced activity levels due to multiple prior surgeries and surrounding soft tissue damage [27]. This clinical context may also decrease shear forces across the construct, further supporting its durability even when used beyond its originally intended temporary purpose.

A potential concern with the use of a metallic component in an infected field is whether it might hinder infection treatment by facilitating biofilm formation. In our cohort, definitive eradication was achieved in 14 of 20 patients (70%). Although the present study lacks a control group and cannot determine whether a metallic liner influences eradication rates, this finding suggests that the use of a cemented DMB metal liner can be compatible with effective surgical debridement and appropriately selected antimicrobial therapy in selected cases. Interpretation of this eradication profile also requires consideration of the microbiological complexity of the study population, which included MRSA in 6 patients (30%), MRCNS in 4 patients (20%), fungal organisms in 2 patients (10%), and polymicrobial infection in 1 patient (5%) (Table 1). These difficult-to-treat pathogens have been associated with lower eradication rates and a higher risk of persistence or relapse across different implant strategies [28,29,30,31]. Accordingly, the three cases of treatment failure (15%) that required resection arthroplasty due to persistent infection, including those involving MRSA and Candida albicans, may more plausibly reflect pathogen-related treatment difficulty rather than a specific detrimental effect of the metallic liner itself. Overall, our data support the feasibility of DMB-incorporated FAHS as a strategy to achieve infection management while preserving joint function in selected complex cases.

This study has several limitations that should be acknowledged. First, its retrospective nature introduces inherent biases, and the relatively small sample size limits statistical robustness and generalizability. In addition, intraoperative selection of this off-label construct based on acetabular defect characteristics may have introduced selection bias, enriching the cohort for more complex cases. Additionally, while the inclusion of both PJI and native hip infections introduces heterogeneity, we believe that combining them can be academically justified on the basis of a shared reconstructive scenario. In advanced native hip infection, acetabular destruction and soft tissue compromise are frequently encountered and can resemble the reconstructive challenges encountered in PJI. Accordingly, the DMB-incorporated FAHS was evaluated as a reconstructive and infection management strategy for compromised infected hips, rather than as a diagnosis-specific intervention. Another limitation is that the absence of comparative groups using conventional PROSTALAC constructs prevents direct evaluation of relative benefits. The present work was intentionally designed as a focused feasibility case series; although inclusion of a comparator group using conventional PROSTALAC designs would have strengthened the analysis, our goal was to determine whether this off-label DMB-incorporated FAHS could be mechanically and clinically acceptable in challenging infection cases. A subsequent comparative study is planned. Finally, functional outcome measures, including patient-reported pain and satisfaction, were not systematically captured, restricting comprehensive evaluation of patient-centric advantages.

## 5. Conclusions

In conclusion, the off-label use of DMB-incorporated FAHS represents a feasible option with acceptable mechanical performance in selected cases of PJI and native hip joint infection, especially in healthcare environments where standardized commercial articulating systems are not readily available. However, as mechanical complications cannot be fully prevented, meticulous surgical technique and careful patient selection remain essential.

## Figures and Tables

**Table 1:** Patient Demographics and Clinical Characteristics.

Variables	Value (Mean ± SD or *n*)
Age (years)	72.9 ± 12.0
Sex	
Men	7
Women	13
BMI (kg/m^2^)	21.6 ± 3.7
Follow-up (months)	23.5 ± 17.4
Diagnosis	
Native hip joint infection	7
Periprosthetic joint infection	13
Identified pathogen	
MSSA	1
MRSA	6
MRCNS	4
* Streptococcus agalactiae*	1
* Escherichia coli*	3
* Candida albicans*	2
Multiple pathogens *	1
No growth	2

SD, standard deviation; BMI, body mass index; MSSA, methicillin-susceptible *Staphylococcus aureus*; MRSA, methicillin-resistant *Staphylococcus aureus*; MRCNS, methicillin-resistant coagulase-negative *Staphylococci*. * Methicillin-susceptible coagulase-negative *Staphylococci*, *Proteus mirabilis*, *Pseudomonas aeruginosa*, and *Vancomycin-resistant enterococci*.

**Table 2:** Descriptive Subgroup Outcomes by Diagnosis.

	PJI (*n* = 13)	Native Hip Infection (*n* = 7)
Infection Control Outcomes		
Infection eradication	9 (69.2%)	5 (71.4%)
Clinical control under chronic suppression	2 (15.4%)	1 (14.3%)
Failure (resection arthroplasty)	2 (15.4%)	1 (14.3%)
FAHS Retention among Eradication Cases	5/9 (55.6%)	2/5 (40.0%)
Any Mechanical Complications	2 (15.4%)	2 (28.6%)
Dislocation	2 (15.4%)	0 (0.0%)
Implant failure	0 (0.0%)	2 (28.6%)

PJI, periprosthetic joint infection; FAHS, functional articulating hip spacer. Data are presented as *n* (%) unless otherwise specified; FAHS retention among eradication cases is presented as *n*/N (%).

## Data Availability

The data presented in this study are available upon request from the corresponding author. The data are not publicly available due to privacy and ethical restrictions related to the sensitive nature of patient medical records and clinical outcomes.

## Abbreviations

The following abbreviations are used in this manuscript:

PJI	Periprosthetic joint infection
FAHS	Functional articulating hip spacer
DMB	Dual-mobility bearing
THA	Total hip arthroplasty
